# Jarcho-Levin Syndrome: Two Consecutive Cases in the Same Family 

**Published:** 2019-12

**Authors:** Patsouras Grigorios, Maroudias George, Saloum Ioannis, Patsouras Konstantinos

**Affiliations:** 1 Department of Obstetrics and Gynecology, “Tzaneio Hospital”, Athens, Greece; 2 Third Department of Obstetrics and Gynecology, "Attikon Hospital", Medical School, National and Kapodistrian University of Athens, Athens, Greece

**Keywords:** Jarcho-Levin Syndrome, Spondylocostal Dysostosis (SCD), Growth Disorder, Genetic Testing

## Abstract

Jarcho-Levin Syndrome was first defined in 1938 by Saul Jarcho and Paul Levin. In the medical literature Jarcho-Levin Syndrome has a variety of synonyms such as Spondylocostal dysplasia/Dysostosis, Spondylocostal Dysostosis (SCD), Spondylothoracic dysplasia/Dysostosis and costovertebral dysplasia. For years the SCD and a similar disorder, spondylothoracic dysplasia, were considered the same disorder and referred as Jarcho-Levin Syndrome. Today we know that these two disorders are different clinical entities with different causes and that the term Jarcho-Levin Syndrome should be reserved for individuals with Spondylocostal dysplasia. Affected individuals with SCD have various abnormalities in the development of the spine and ribs. Due to these abnormalities they are more prone to develop thoracic insufficiency syndrome which may eventually lead to early neonatal death. In the current case report we describe two consecutive cases with SCD in the same family. In the case of a strong clinical suspicion from the findings of the ultrasound scan we should proceed to a molecular genetic diagnosis of a mutation in DLL3, MESP2, LFNG and HES7 gene by sequencing the entire coding area of the fetal Deoxyribonucleic acid (DNA).

## Introduction

Spondylocostal Dysostosis (SCD) or Jarcho-Levin Syndrome is a rare hereditary growth disorder. It was first described in 1938 by Saul Jarcho and Paul Levin (1). They published a study about two siblings with thoracic insufficiency due to multiple vertebral and rib malformations (1). Since then more than 130 cases have been reported worldwide. In the medical literature Jarcho-Levin Syndrome has a variety of synonyms such as Spondylocostal dysplasia/Dysostosis, SCD, Spondylothoracic dysplasia/Dysostosis (STD) and costovertebral dysplasia (2). For years there has been a significant confusion in the medical literature regarding these synonyms. The SCD and a similar disorder, spondylothoracic dysplasia, were considered the same disorder and referred as Jarcho-Levin Syndrome. Many doctors were using the ‘umbrella’ term, Jarcho-Levin Syndrome, for various conditions associated with spinal and rib defects (3). Today we know that these two disorders are different clinical entities with different causes and that the term Jarcho-Levin Syndrome should be reserved for individuals with Spondylocostal dysplasia (3). It is characterized by congenital defects of the vertebrae which may be fused together or missing or are underdeveloped and wedge-shaped (hemivertebrae) (4). Another characteristic finding is the presence of congenital malformations of the ribs, which may be absent or fused or overgrowth (4). In the fast majority, the SCD is inherited by an autosomal recessive manner due to a mutation in one of four genes DLL3, MESP2, LFNG, HES7 of the Notch pathway genes. Rarely it can be inherited by an autosomal dominant way due to a mutation in the TBX6 gene. Nevertheless many individuals do not have a mutation in any of the abovementioned genes. Spondylocostal dysplasia equally affects both men and women in a panethnic manner (4). Affected individuals have various abnormalities in the development of the spine and ribs. Due to these abnormalities they are more prone to develop thoracic insufficiency syndrome which may eventually lead to early neonatal death. Despite these serious complications, approximately 50% of all cases will live until the adulthood (5). The SCD can be diagnosed prenatal during the anatomy scan in the second trimester. The characteristic ultrasound findings are: vertebral defects (such as hemivertebrae) or ribs malformations which will have a fan-shaped or a crab-like appearance (6).

## Case Presentation

**Figure 1:**
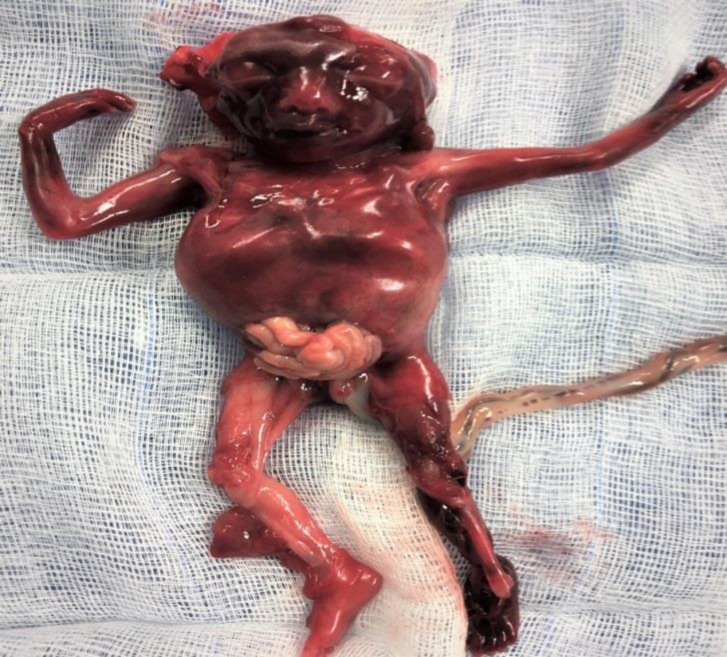
The fetus with the placenta after the induced abortion. Note that the arms and legs are normal but the thorax is foreshortened and ribs are fused (Salloum 2017)

We present the case of a 34 year-old pregnant woman G^3^P^2^A^0^L^2^ from Syria, who was referred to our department at 14 weeks gestational age (02/2017) for evaluation of an abnormal first trimester nuchal translucency ultrasound scan including an abnormal spine (fused and underdeveloped vertebra) and a gastroschisis. The rest of her blood exams from her first antenatal visit were normal. From her past obstetrical history, she mentioned two term vaginal deliveries 9 and 11 years ago. From the maternal or the paternal past medical history there were no predisposing risk factors such as smoking or alcohol consumption. There was no family history of dwarfism and the patient and her partner, who was from Livanos, were non-consanguineous. During the detailed scan, we confirmed the abovementioned ultrasound findings. So we consult the couple to proceed with Chorionic villus sampling (CVS). The first results from the molecular karyotype came back negative for any of the 512 examined genetic diseases. Due to the specific ultrasound findings, we proceed to a more specific examination in order to reveal any mutation in the DLL3, MESP2, LFNG and HES7 gene. The result was positive for a mutation in the DLL3 gene. After careful and detailed consultation with the couple, they decided to proceed to induced abortion which was successfully completed one week later without any complications (Figure.1). The fetus and the placenta were sent for histopathological examination. The final autopsy report confirmed the presence of various congenital malformations of the spine and ribs, which are characteristic phenotypic findings of the SCD. 

**Figure 2:**
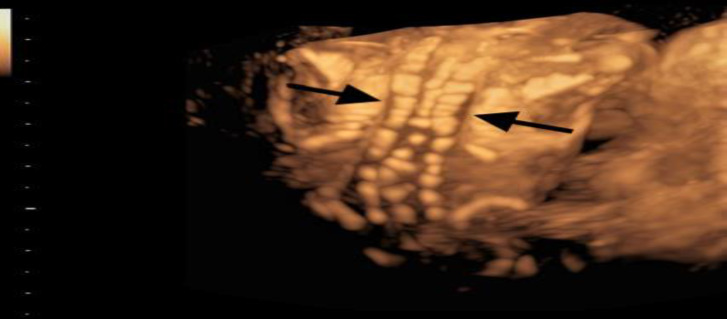
3D view of the fetal spine. Note the characteristic congenital defects of the vertebrae (Salloum 2018)

**Figure 3:**
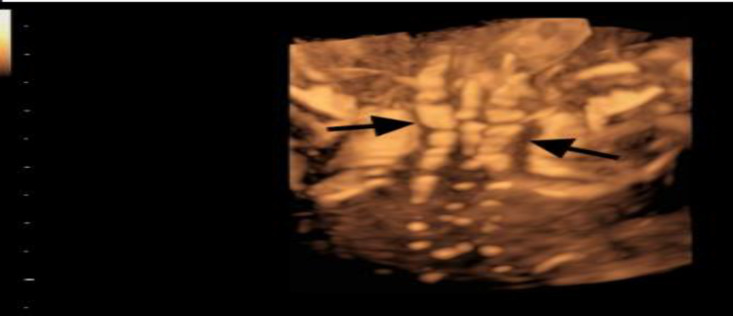
3D view of the fetal spine. Note the characteristic congenital defects of the vertebrae (Salloum 2018)

**Figure 4:**
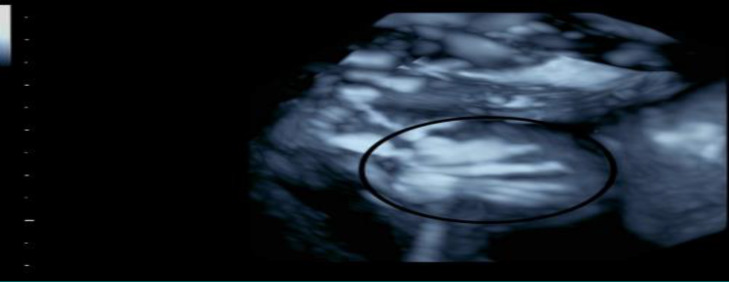
3D view of the fetal thorax. Note the various fused ribs (Salloum 2018)

After the abortion the couple received a specific genetic counseling regarding the recurrence risk. The risk of having an affected offspring is 50%. Ten months later the same couple came to our department for a scheduled first trimester scan which revealed a foreshortened spine with fused and undergrowth vertebrae and multiple fused ribs (Figures 2, 3, 4).

**Figure 5:**
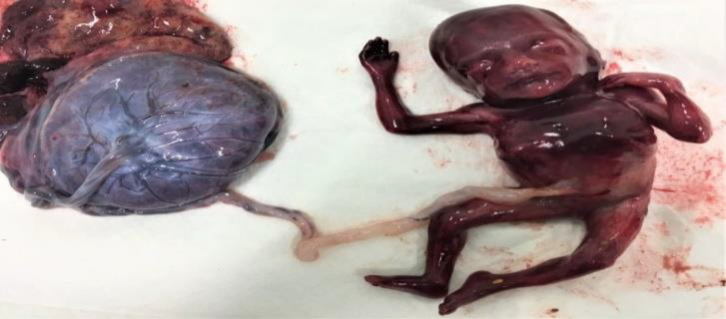
The fetus with the placenta. Note the characteristics phenotypic findings of the Jarcho-Levin Syndrome (Salloum 2018)

So we proceed to CVS. After sequencing the entire coding area of the fetal Deoxyribonucleic acid (DNA) obtained from CVS, we confirmed the presence of a DLL3 gene mutation. The diagnosis of SCD was confirmed for a second time and we proceed to an induced abortion two weeks later (Figure 5).

## Discussion

In the case of a strong clinical suspicion from the findings of the ultrasound scan we should proceed to a molecular genetic diagnosis of a mutation in any of the four abovementioned genes by sequencing the entire coding area of the fetal DNA.

Only then we will have a definitive diagnosis of the SCD and we can continue to detailed genetic counseling of the couple. For years the term Jarcho-Levin Syndrome was inaccurately applied to any offspring affected with either SCD or STD. If eponyms must be used, as they commonly are, it is not justified to continue to apply the ‘umbrella’ term Jarcho-Levin Syndrome to patients with characteristics findings of STD.

## Conclusion

Only after molecular genetic diagnosis of a mutation by sequencing the entire coding area of the fetal DNA we will have a definitive diagnosis of the SCD and we can continue to detailed genetic counseling of the couple.

If eponyms must be used, as they commonly are, it is not justified to continue to apply the ‘umbrella’ term Jarcho-Levin Syndrome to patients with characteristics findings of STD.
